# Considering clinical history as a determinant of human neuron function

**DOI:** 10.1093/braincomms/fcae366

**Published:** 2024-10-12

**Authors:** Sam A Booker

**Affiliations:** Simons Initiative for the Developing Brain, University of Edinburgh, Edinburgh EH8 9XD, UK; Patrick Wild Centre for Research into Autism, Fragile X Syndrome & Intellectual Disabilities, University of Edinburgh, Edinburgh EH8 9XD, UK; Centre for Discovery Brain Sciences, University of Edinburgh, Edinburgh EH8 9XD, UK

## Abstract

This scientific commentary refers to ‘Clinical parameters affect the structure and function of superficial pyramidal neurons in the adult human neocortex’, by Lenz *et al*. (https://doi.org/10.1093/braincomms/fcae351).

**This scientific commentary refers to ‘Clinical parameters affect the structure and function of superficial pyramidal neurons in the adult human neocortex’, by Lenz *et al*. (https://doi.org/10.1093/braincomms/fcae351).**

Paraphrasing Rosenblueth and Wiener,^1^ the best material model of a human is a human, ideally the same human. Such thinking is increasingly important when we consider translating neuroscience findings from model organisms to humans and may help to improve preclinical validation of new therapies from laboratory to bedside. The recent study from Lenz *et al*.^2^ in *Brain Communications* aims to address this credo, by performing a detailed analysis of human neocortical neurons, comparing individuals with and without a clinical history of epilepsy. Specifically, they find that intra-individual variability of neuronal excitability and morphology is high. Furthermore, they show dendritic spine density, while the variable displays age-dependent reductions. They found that a history of epilepsy does not lead to significant modification of neuronal structure and excitability, albeit with a modest sample size. These data should serve as a call to arms for those performing electrophysiological recordings from human brain tissue to better understand the tissue we are working with and utilize these clinical features to provide translational insights to these conditions.

The increased use of human brain tissue has opened up the possibility of generating data to the specific functional diversity and specialization of human cortical neurons^3-5^ and generation of disease models.^6,7^ However, at the core of such endeavours, it is critical to consider the underlying clinical features of the individuals that samples are obtained from. Neurosurgical samples are rarely (if ever) obtained from individuals who have no underlying health conditions. Indeed, most samples are obtained from an epileptogenic focus, access tissue to remove deep-seated brain tumours or introduction of a ventricular shunt.^2-5,7,8^ While even when tumour-free access tissue is used as control and thus assumed to be largely devoid of neuropathology, the present study highlights that these patients often have complex pharmacological histories, which includes: steroids, chemotherapeutics, anti-seizure medications, and importantly intra-operative anaesthetics and muscle relaxants. While it is hoped that the preparation of acute brain slices introduces significant washing and dilution of these compounds from the extracellular space, it is hard to believe that prolonged exposure to such potent modulators of neuronal function does not leave an indelible mark on the functional properties of neurons.^5^ This is likely an even greater confound in epileptic tissues, as neurosurgical resection is often the last option available to patients. Subsequently, tissue from the seizure focus is not only the source of seizure activity, but the patients it has been obtained from have often received combinatorial drug therapies for years prior to surgery. Interpreting the differences in neuron function and resident cell types in the cortex following prolonged drug/seizure exposure could introduce significant confounds. Therefore, it is somewhat surprising that Lenz *et al*.^2^ did not see greater differences between clinical groups. However, aspects of their data are consistent with previous studies in resected human tissue, where individuals prescribed anti-seizure medications displayed minimal differences in tonic GABA conductance^4^ but may display altered metabotropic GABA_B_ receptor signalling,^5^ which was not tested here.

A considerable strength of this study is that the authors sought to compare physiological function and anatomical parameters in the same cells, performing correlative analyses both of cellular features and age. Indeed, Lenz *et al*.^2^ show that there is a high intra-individual variability in cellular properties that correlate with age—from a mixed population of Layer 2 and 3 human neurons. Recent studies have revealed that in the human neocortex, five major classes of pyramidal cell exist.^3^ How much these different cell types contribute to the variability shown here and how they change their features as a function of age require further extensive study. Furthermore, examination of dendritic spine properties in this study shows minimal change in dendritic spine head diameter over age, which is contrary to previous reports.^9^ Such difference may arise from the smaller biological samples of this earlier study or indeed that both of these current studies rely on diffraction-limited imaging, which may introduce extraneous experimental variability to the resulting measurements.^10^ Future studies employing *post hoc* super-resolution imaging combined with correlation of functional properties such as synaptic strength and cellular diversity would give us unparalleled insights into the functional diversity of human cortical circuits in health and disease. Only by understanding how cellular diversity contributes to pathological and non-pathological aging in humans will we be able to make translational comparisons with our extensive knowledge of these processes in rodents.

Overall, Lenz *et al*.^2^ highlight the critical importance of understanding from whence we obtain human tissue samples. Such live tissue samples represent a hugely important bioresource for neuroscience research; however, drawing inferences of function from variable samples from different life stages may give rise to conclusions that fail to faithfully reflect underlying biological diversity and processes. Implementing cutting-edge imaging and analytical approaches from ever-increasing sample sizes across the adult life span will no doubt open more questions about the function of the brain but hopefully also begin to understand the cellular and circuit features that define the human condition.

## Data Availability

No data was generated for this commentary.
